# New vaccines to prevent otitis media

**DOI:** 10.1016/S1808-8694(15)30645-5

**Published:** 2015-10-19

**Authors:** Tania Maria Sih, Lucia Ferro Bricks

**Affiliations:** 1 PhD. Professor – FMUSP – University of São Paulo Medical School, Medical Investigations Lab (MIL) # 40; 2 PhD in Medicine – FMUSP – Professor – Department of Pediatrics – USP

**Keywords:** otitis media, vaccines

AC/ Fernando Felix

Ref Artigo de Felix et al (2008)

After the publication of the excellent review paper on vaccines to prevent otitis media, by Felix et al. (2008) ^1^, we found new data on the relationship between acute respiratory tract infections (ARTI) caused by virus and otitis media in children, which we believe to be relevant for the readers of this well regarded journal 2, 3, 4, 5, 6.

Chonmaitree et al.^2^ isolated viruses from children with ARTI, and more than half of the 864 episodes of viral ARTI (61%) were complicated by acute otitis media: 37% (AOM) and 24% with otitis media with effusion (OME). Yano et al.^3^ studied 1,092 children with ARTI followed by AOM, and they isolated viruses from the respiratory secretion and the middle ear fluid and noticed that the highest rates of viral isolates were obtained when the tympanic membrane punction (tympanocentesis) was carried out at the early onset of symptoms in infants with fever. Therefore, in infections which the material collection happened later on, it was not possible to isolate the agent responsible for the alterations that caused the secondary bacterial infection.

Revai et al.^5^ assessed the alterations caused by ARTI in previously healthy children aged between 6 and 35 months, and noticed that 1/3 of the children with age between 6 and 24 months had alterations in their tympanometries during the first week of these infections. The alterations were significantly more frequent and severe when compared to the ones seen in children older than two years and, according to the authors, these alterations can explain the greater incidence of AOM secondary to viral infections at this age range.

The prevention of viral and bacterial acute respiratory tract infections through the use of influenza vaccines and tandem vaccines against S. pneumoniae can substantially reduce the incidence of AOM and OME and, consequently, the use of antibiotics, the selection and spread of bacterial strains resistant to antibiotics, as well as the costs and risks associated with unnecessary treatment^1^. Recently, Jansen et al.^6^ did a randomized, double blind and controlled study in which the use of influenza vaccine in children, with or without the concomitant administration of the 7-valient tandem vaccine against pneumococcus (PV 7), reducing in half the incidence of otitis for a period longer than the seasonality of influenza infections (winter time).

Both the PV 7 and the vaccine against influenza are recommended by the Brazilian Association of Pediatrics for all healthy children. PV 7 is recommended for children older than six months and also for family members and people who have contact with children younger than two years, including family members, care takers and health care professionals.

We stress the need to give two doses of influenza vaccine in the prime-vaccination of children aged between six months and nine years, and we stress that its efficacy varies according to the strain of the season, age, immune status and the presence of associated risks.

The influenza vaccine is safe and its use is capable of reducing up to 50% of the cases of post-influenza AOM, besides preventing other more severe complications, such as pneumonia, hospitalization and death. Influenza vaccine coverage is still low in children, even in risky groups such as asthma patients, for whom the vaccine is freely available from public health care facilities. Most non-vaccinated people, including those in greater risks, were not vaccinated for lack of medical advice. Physicians are not only responsible for treating diseases, but they must also educate the population on the best way to avoid them.
